# On-Membrane Dynamic Interplay between Anti-GM1 IgG Antibodies and Complement Component C1q

**DOI:** 10.3390/ijms21010147

**Published:** 2019-12-24

**Authors:** Saeko Yanaka, Rina Yogo, Hiroki Watanabe, Yuki Taniguchi, Tadashi Satoh, Naoko Komura, Hiromune Ando, Hirokazu Yagi, Nobuhiro Yuki, Takayuki Uchihashi, Koichi Kato

**Affiliations:** 1 Exploratory Research Center on Life and Living Systems (ExCELLS) and Institute for Molecular Science (IMS), National Institutes of Natural Sciences, 5-1 Higashiyama, Myodaiji, Okazaki, Aichi 444-8787, Japan; saeko-yanaka@ims.ac.jp (S.Y.); yogo@ims.ac.jp (R.Y.); hwatanabe@ims.ac.jp (H.W.); 2 Faculty and Graduate School of Pharmaceutical Sciences, Nagoya City University, 3-1 Tanabe-dori, Mizuho-ku, Nagoya, Aichi 467-8603, Japan; yuuki82540@gmail.com (Y.T.); tadashisatoh@phar.nagoya-cu.ac.jp (T.S.); hyagi@phar.nagoya-cu.ac.jp (H.Y.); 3 Center for Highly Advanced Integration of Nano and Life Sciences (G-CHAIN), Gifu University, Yanagido 1-1, Gifu 501-1193, Japan; komura@gifu-u.ac.jp (N.K.); hando@gifu-u.ac.jp (H.A.); 4 Yuki Clinic, 1-3-7 Johnan, Mito, Ibaraki 310-0803, Japan; gbs.yuki.cidp@gmail.com; 5 Department of Physics, Nagoya University, Furo-cho, Chikusa-ku, Nagoya, Aichi 464-8602, Japan

**Keywords:** high-speed atomic force microscopy, immunoglobulin G, Fc, ganglioside GM1, Guillain–Barré syndrome, protein A

## Abstract

Guillain–Barré syndrome, an autoimmune neuropathy characterized by acute limb weakness, is often preceded by *Campylobacter jejuni* infection. Molecular mimicry exists between the bacterial lipo-oligosaccharide and human ganglioside. Such *C. jejuni* infection induces production of immunoglobulin G1 (IgG1) autoantibodies against GM1 and causes complement-mediated motor nerve injury. For elucidating the molecular mechanisms linking autoantigen recognition and complement activation, we characterized the dynamic interactions of anti-GM1 IgG autoantibodies on ganglioside-incorporated membranes. Using high-speed atomic force microscopy, we found that the IgG molecules assemble into a hexameric ring structure on the membranes depending on their specific interactions with GM1. Complement component C1q was specifically recruited onto these IgG rings. The ring formation was inhibited by an IgG-binding domain of staphylococcal protein A bound at the cleft between the C_H_2 and C_H_3 domains. These data indicate that the IgG assembly is mediated through Fc–Fc interactions, which are promoted under on-membrane conditions due to restricted translational diffusion of IgG molecules. Reduction and alkylation of the hinge disulfide impaired IgG ring formation, presumably because of an increase in conformational entropic penalty. Our findings provide mechanistic insights into the molecular processes involved in Guillain–Barré syndrome and, more generally, into antigen-dependent interplay between antibodies and complement components on membranes.

## 1. Introduction

Immunoglobulin G (IgG) is a crucial mediator of the defensive mechanisms for eliminating infectious microorganisms [1]. Antigenic determinants displayed on the surfaces of invasive cells are recognized by host IgG antibodies, which trigger effector functions, such as complement-mediated cytotoxicity and opsonic phagocytosis. To evade the host immune system, mechanisms have evolved in infectious bacteria to coat their surfaces with molecules derived from infected hosts or molecules mimicking those presented on host cells. Some Gram-positive bacteria possess cell wall proteins capable of binding to IgG molecules independent of their antigen-binding sites. Examples of such proteins are protein A from *Staphylococcus aureus* and protein G from streptococcus groups C and G, which can disturb host immune mechanisms [2,3]. In contrast, some Gram-negative bacteria can express outer membrane glycolipids that share common glycan structures with mammalian glycosphingolipids, enabling them to escape the immune surveillance [4,5]. However, such bacterial glycolipids occasionally elicit the production of antibodies that are cross-reactive with host molecules [6]. Therefore, the molecular mimicry between components of infectious bacteria and the host has been postulated as the mechanism underlying the onset and development of autoimmune diseases.

Guillain–Barré syndrome is a post-infectious autoimmune neuropathy characterized by acute limb weakness. One-third of patients with Guillain–Barré syndrome are preceded by *Campylobacter jejuni* enteritis [7]. Molecular mimicry exists between two-thirds of *C. jejuni* strains and human ganglioside GM1, which is a glycosphingolipid highly expressed at the nodal membranes of human motor nerves. In one out of one thousand individuals, such *C. jejuni* infection could induce the production of anti-GM1 IgG1 autoantibodies and consequent complement-mediated motor nerve injury, causing limb weakness [8]. However, the molecular mechanisms linking autoantigen recognition and complement activation remain largely unknown.

To gain mechanistic insights into the molecular process behind these interactions, we attempted to observe the dynamic interactions of an anti-GM1 monoclonal autoantibody, GB2. This autoantibody was generated by immunization of mice with GM1-like lipo-oligosaccharide purified from a *C. jejuni* strain isolated from a patient with Guillain–Barré syndrome [6]. In a previous study, we demonstrated that high-speed atomic force microscopy (HS-AFM) is a powerful tool for real-time observation of interactions of IgG molecules with the Fcγ receptor [9]. Here we applied this technique for visualizing the interplay between GB2 and complement component C1q on membranes containing GM1, which is the first step of the classical complement pathway in the immune system.

## 2. Results

### 2.1. Epitope Mapping of GB2

**Figure 1:**
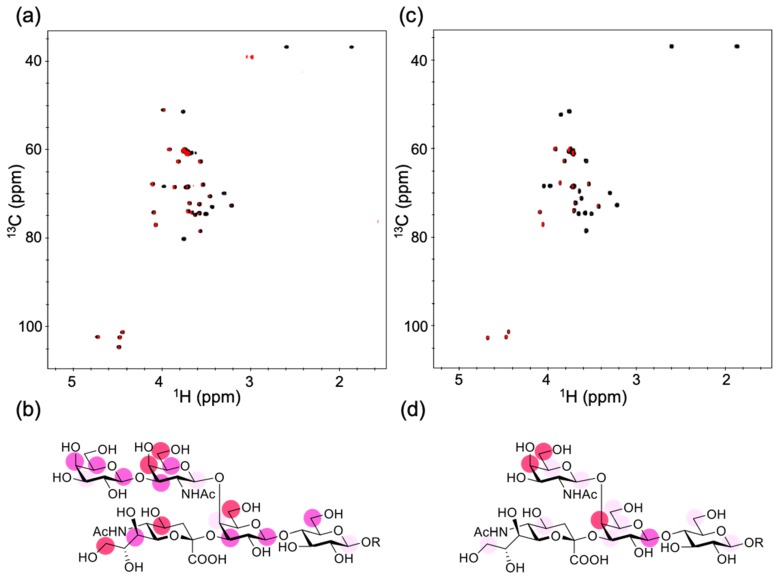
NMR observation of the interaction of gangliosidic oligosaccharides with GB2. (**a**) Overlay of the 2D ^1^H–^13^C heteronuclear single-quantum correlation (HSQC) spectrum (black) and the ^1^H–^13^C saturation transfer difference (STD)-HSQC spectrum (red) of the 2-trimethylsilylethyl derivatives of the GM1 pentasaccharide (10 equiv.) with 50 μM GB2. (**b**) Mapping of the binding epitope of the GM1 pentasaccharide. (**c**) Overlay of the 2D ^1^H–^13^C HSQC spectrum (black) and the ^1^H–^13^C STD-HSQC spectrum (red) of the 2-trimethylsilylethyl derivatives of the GM2 tetrasaccharide (10 equiv.) with 50 μM GB2. (**d**) Mapping of the binding epitope of the GM2 tetrasaccharide. In (**b**,**d**), the maximum peak intensity of each oligosaccharide was taken as 100%, and the relative intensities (over 80% red, over 65% pink, over 50% light pink) are indicated. R represents a 2-trimethylsilylethyl group.

Previous studies revealed that GB2, directed against *C. jejuni*, was cross-reactive with GM1 but not with the other ganglioside components in the bovine brain, e.g., GD1a, GD1b, and GT1b [6], nor synthesized gangliosidic oligosaccharides derived from GM3, GD3, GM2, GD1a, and GT1a [10]. We conducted a saturation transfer difference (STD) NMR experiment using a synthetic GM1 pentasaccharide for characterizing antigen recognition by GB2. In this experiment, the saturated magnetization of antibody protons can be transferred to the oligosaccharide in their bound state, thereby enabling the identification of the carbohydrate epitope recognized by GB2 based on STD data. The STD data indicated that the common carbohydrate moiety shared with *C. jejuni* lipo-oligosaccharide was extensively involved in the interaction with the antibody GB2, which explained its highly specific interaction with GM1 (Figure 1a,b). Our STD data obtained by using a GM2 tetrasaccharide also confirmed that the interaction was significantly compromised by the removal of the outer galactose residue. In the subsequent experiments, we characterized the interaction of GB2 with GM1 in membrane environments (Figure 1c,d).

### 2.2. IgG Assembly on Antigen-Incorporated Membranes

**Figure 2:**
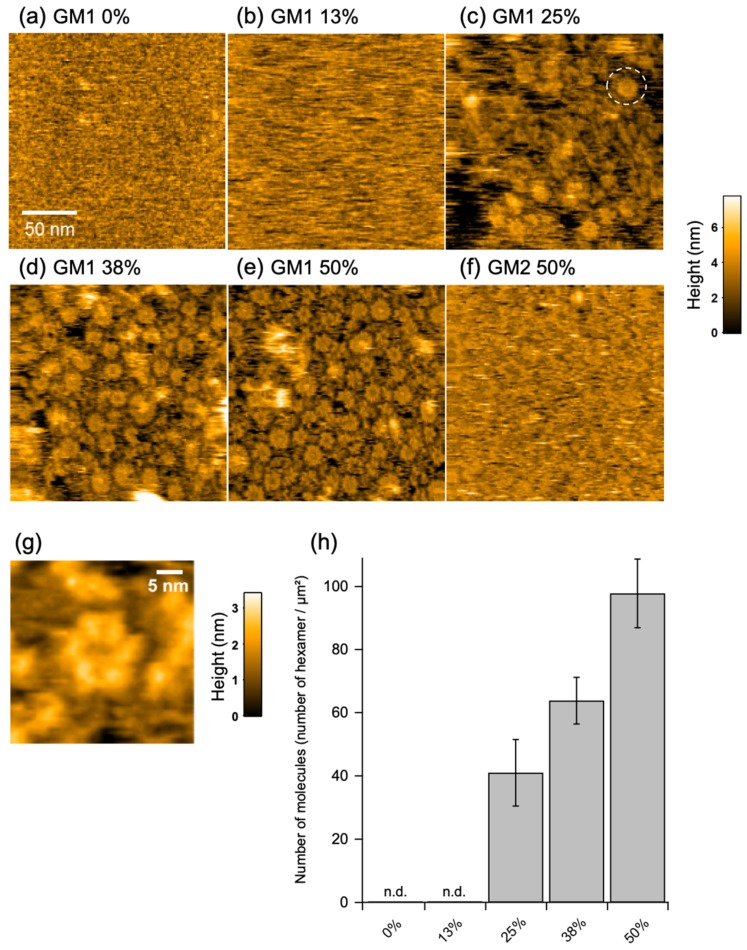
High-speed atomic force microscopy (HS-AFM) observation of assembly of GB2 on membranes. HS-AFM images of immunoglobulin G (IgG) on the GM1-incorporated 1,2-dioleoyl-sn-glycero-3-phosphocholine (DOPC) membranes containing (**a**) 0%, (**b**) 13%, (**c**) 25%, (**d**) 38%, and (**e**) 50% GM1 and (**f**) 50% GM2. A typical IgG hexameric ring is indicated by dotted circles. (**g**) A high-resolution image of IgG hexamer. (**h**) Summary of the number of IgG hexamers counted in the HS-AFM images.

For HS-AFM observation of the molecular behavior of GB2 on a membrane, a mica surface was covered with a lipid bilayer composed of 1,2-dioleoyl-sn-glycero-3-phosphocholine (DOPC) and GM1 in varying ratios. The GM1/DOPC lipid had a uniform surface but not phase-separated domains (Supplementary Figure S1a). In addition, we confirmed that GM1 molecules were uniformly distributed in the membrane as probed with cholera toxin B subunit, a GM1-specific binder (Supplementary Figure S1b). In the absence of GM1, we could not see obvious spots of the IgG molecules bound to the membrane. This is because the affinity of IgG for DOPC was so weak that IgG diffused much faster than the imaging speed of HS-AFM. By contrast, binding of the IgG molecules assembled into well-ordered hexameric ring structures, besides larger aggregates and assembly intermediates, was clearly observed on the membranes with higher (>25%) GM1 ratios (Figure 2, Supplementary Movies S1–S3). When GM2 was used instead of GM1, the IgG molecules flitted on the membrane but never formed the ring structure (Figure 2f, Supplementary Movie S4). These results indicate that antigen binding promotes the hexameric ring formation of the GB2 antibodies on the membrane.

### 2.3. C1q Interaction with the IgG Hexameric Ring Formed on Membranes

**Figure 3:**
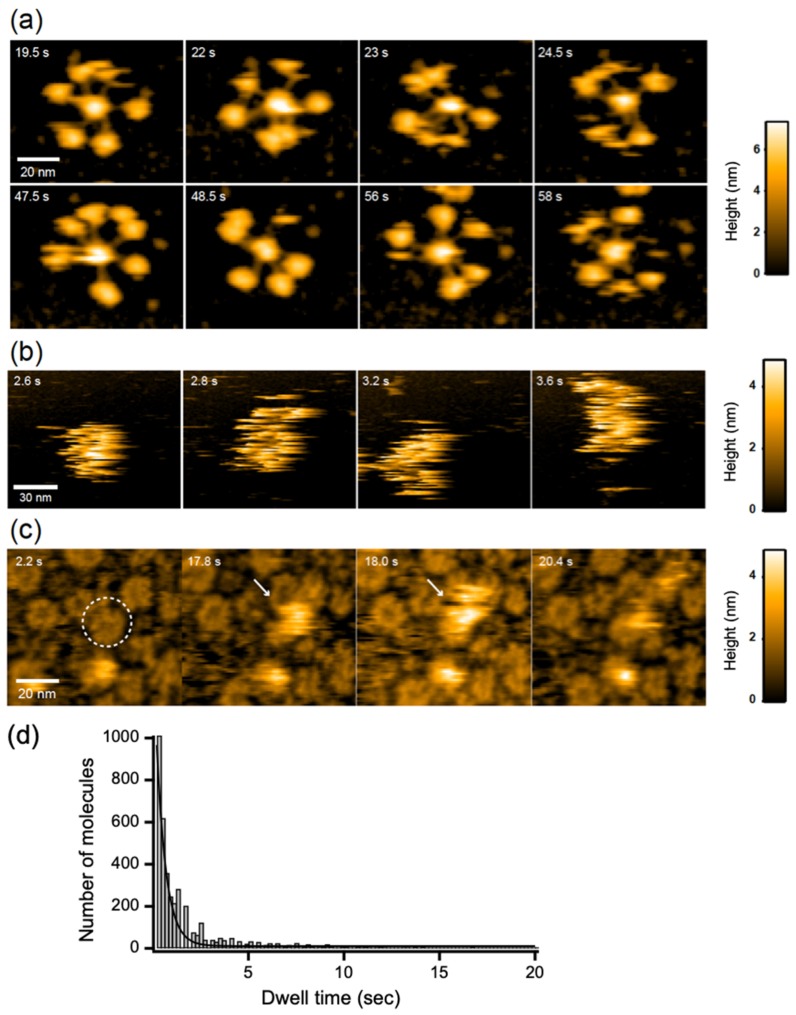
HS-AFM observation of C1q. C1q on (**a**) mica surface and DOPC membranes containing 50% GM1 in (**b**) the absence and (**c**) presence of GB2. A typical IgG hexameric ring is indicated by dotted circles. The C1q bound to the IgG ring is indicated by white arrows. (**d**) The dwell time of C1q on the IgG hexameric ring formed on the GM1-incorporated membrane.

Next, we attempted to address the pathological relevance of the antigen-dependent IgG hexamer formation. We examined the potential interaction of the IgG hexameric ring with C1q using HS-AFM, because accumulating data have indicated that C1q preferentially binds to IgG oligomers [11,12,13,14]. HS-AFM could clearly visualize dynamic structures of free C1q molecules on mica surface treated with 3-aminopropyltriethoxysilane, exhibiting six highly mobile globular heads tethered to the central stem, whereas C1q molecules were not clearly observable on the membranes because of rapid diffusion due to weak interaction with the membrane surface (Figure 3a,b, Supplementary Movies S5 and S6). Intriguingly, in the presence GB2, C1q often visited the hexameric rings formed by this antibody with a residence time of 0.49 ± 0.03 s (Figure 3c,d, Supplementary Movie S7).

### 2.4. Effect of Protein A Binding and Disulfide Cleavage on IgG Hexameric Ring Formation

**Figure 4:**
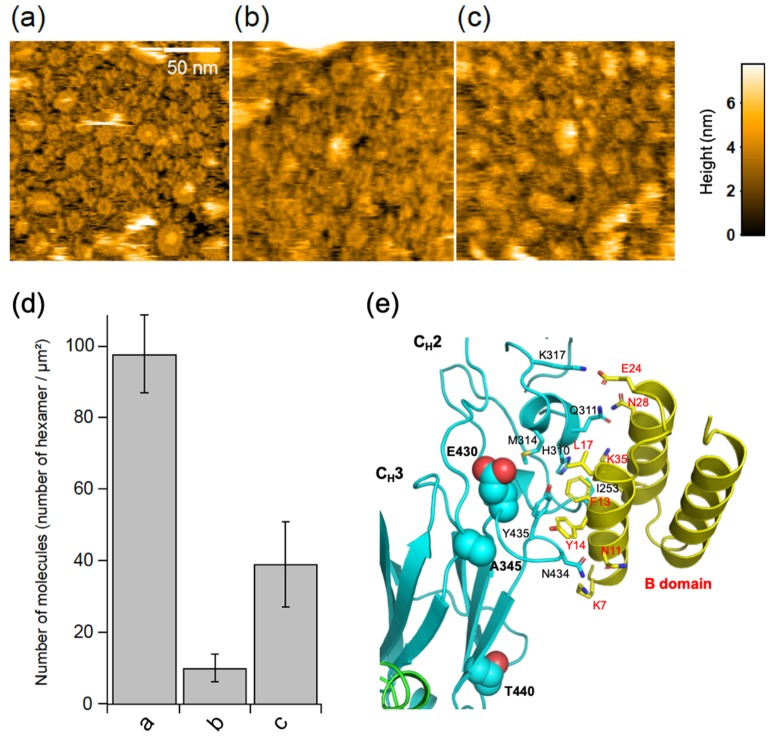
Effects of the B domain of protein A and hinge disulfide cleavage on IgG ring formation. HS-AFM images of GB2 in the (**a**) absence or (**b**) presence of the B domain of protein A or (**c**) reduced and alkylated GB2 on the 50% GM1-incorporated DOPC membranes. (**d**) Summary of the number of IgG hexamers in each treatment. Each bar corresponds to the data obtained under the conditions of a, b, or c. (**e**) X-ray crystallographic structure of Fc interacting with B domain of protein A. Residues involved in the interaction are shown as sticks, whereas the corresponding residues in the human Fc that were mutated to enhance hexamer formation are shown as balls.

The bacterial IgG-binding proteins, protein A and protein G, have been reported to inhibit C1q binding to IgG antibodies [2,15]. Our HS-AFM data showed that GB2 scarcely formed the hexameric ring structure in the presence of the B domain of protein A, which is known to bind the Fc region of IgG antibodies [2,16,17,18] (Figure 4). We solved the crystal structures of the Fc fragment of GB2 and that of the same IgG isotype in the absence or presence of the B domain of protein A, respectively. In the crystal, the apo form of Fc formed a trimer but not a hexamer, confirming that the ring formation is an on-membrane process mediated by antigen-Fab interaction (Supplementary Figure S2). Our crystallographic data also confirmed that, as in the case of previously reported crystal structures [17,18], the Fc interacts with the B domain of protein A at the cleft between the C_H_2 and C_H_3 domains, which is also involved in protein G binding [19,20] (Figure 4e, Supplementary Figure S3). These data indicate that the antigen-dependent hexameric ring formation of IgG is mediated by Fc–Fc interactions, which is disrupted by competitive binding with protein A.

Complement activation was reported to be impaired by the reduction and alkylation of disulfide bonds in the hinge region of IgG antibodies [21]. We examined the possible impact of the cleavage of IgG interchain disulfide bridges on the antigen-dependent assembly of the GB2 autoantibodies. The results indicate that the disulfide cleavage causes a significant reduction in the GB2 hexamer formation (Figure 4c,d).

## 3. Discussion

The anti-GM1 antibody GB2 was found to stain nodes and axons in the motor nerve roots in rabbit models of Guillain–Barré syndrome [6]. Anti-GM1 IgG antibodies cause complement-mediated membrane disruption at nodes of Ranvier in peripheral motor nerve fibers in humans and rabbits [22,23]. Our study visualized antigen-dependent assembly of the GB2 antibodies on model membranes, giving rise to hexameric ring structures, which particularly interacted with C1q.

In recent studies, the binding of C1q with IgG oligomers was studied, most notably by employing artificially hexamerized IgG antibodies [11,12,13,14]. It has been reported that IgG molecules have the potential ability to form hexamers in crystal or on mica surface under certain conditions [14,24]. In human IgG, the hexamer formation could be enhanced by triple mutation at the C_H_3 domain (i.e., E345R, E430G, and S440Y), which improves the complement activating activity [14]. Cryo-electron microscopy data have revealed that C1q interacts with the hexamerized IgG, and each globular head of C1q is bound to the hinge-proximal C_H_2 domains of IgG antibodies, which is distal from the binding sites of the bacterial IgG-binding proteins [12]. Intriguingly, the mouse anti-GM1 IgG2b(κ) antibody GB2, in which positions 345, 430, and 440 are occupied by alanine, glutamic acid, and threonine, respectively, were highly preorganized into the hexameric ring on the antigenic membrane formation without artificial mutations. These amino acid residues are not directly involved in the binding to the B domain of protein A, which, however, sterically inhibits Fc–Fc interaction. Therefore, the inhibition of C1q binding by protein A and protein G is, at least, partially attributed to the prevention of Fc-mediated ring formation.

In the wild-type IgG2b, the hexameric ring formation occurred exclusively on the membranes containing specific antigenic molecules and did not occur in solution. This ordering process is entropically unfavorable; therefore, it is promoted selectively under on-membrane conditions, where diffusion of IgG molecules is considerably restricted. Upon loss of the constraint of disulfide bonds at the hinge, IgG antibodies gain greater degrees of motional freedom of the C_H_2 domains, with an increase in the population of extremely asymmetric quaternary conformations [25]. Therefore, the reduction and alkylation of the hinge disulfides of IgG causes an increase in the conformational entropic penalty for hexamerization, resulting in the impaired formation of IgG rings reactive with C1q.

In summary, our findings provide mechanistic insights into the molecular processes involved in Guillain–Barré syndrome and, more generally, into the antigen-dependent interplay between antibodies and complement components on membranes. It is possible that Fc-mediated hexameric ring formation depends not only on antigen type but also on IgG glycoforms because C1q binding to IgG is reported to depend on galactosylation and sialylation of Fc [26,27,28]. Our subsequent studies will address these issues, which will provide clues to control IgG assembly and the consequent complement activation by employing antibody engineering approaches.

## 4. Materials and Methods

### 4.1. Chemicals

GM1, GM2, and DOPC were purchased from Avanti Polar (Alabaster, AL, USA). The GM1 pentasaccharide and the GM2 tetrasaccharide were synthesized as 2-trimethylsilylethyl glycoside derivatives from the reported *O*-acyl protected precursors according to the standard saponification method [29,30].

### 4.2. Protein Preparation

#### 4.1.1. Antibody

The mouse anti-GM1 IgG2b(κ) antibody GB2 was produced in mouse hybridoma cells [6], which were cultivated in the NYSF 404 serum-free medium (Nissui, Tokyo, Japan). After cell growth, the medium supernatant was applied onto an nProtein A Sepharose Fast Flow column (GE Healthcare, Chicago, IL, USA), followed by gel filtration using a HiLoad 16/60 Superdex 200 pg column (GE Healthcare, Chicago, IL, USA) with phosphate-buffered saline consisting of 137 mM NaCl, 2.7 mM KCl, 8.1 mM Na_2_HPO_4_, and KH_2_PO_4_ (pH 7.4) to purify GB2. Cleavage of the interchain disulfide bridges of GB2 was performed according to the literature [31]. For brevity, GB2 was reduced by 10 mM DTT at room temperature for 1 h in 1.5 M Tris-HCl, pH 8.5, containing 2 mM EDTA. For alkylation, 22 mM iodoacetic acid was added to the above reaction mixture, which was incubated in the dark for 20 min at room temperature. The GB2 antibody and its reduced and alkylated analog, thus prepared, were dialyzed against phosphate-buffered saline and were subjected to HS-AFM measurements. For X-ray crystallographic analysis, the Fc fragments were prepared from GB2 and mouse anti-progesterone IgG2b(κ) antibody 7D7 [32] by papain digestion, performed at 37 °C for 12 h in 75 mM sodium phosphate buffer (pH 7.0) containing 75 mM NaCl and 2 mM EDTA. The protein concentration was 10 mg/mL, and the ratio of papain:IgG was 1:50 (w:w). The digestion products were loaded onto an Affi-gel protein A column (Biorad, Berkely, CA, USA) to obtain purified Fc fragments.

#### 4.1.2. Protein A

The cDNA of the B domain of protein A was purchased from Fasmac Co., Ltd. (Kanagawa, Japan) and subcloned into a pET28b expression vector (Merck KGaA, Darmstadt, Germany). A recombinant B domain with an *N*-terminal hexahistidine tag was expressed in *Escherichia coli* strain BL21(DE3)-CodonPlus (Stratagene, San Diego, CA, USA). The hexahistidine-tagged B domain was then purified using a Chelating Sepharose Fast Flow column (GE Healthcare, Chicago, IL, USA). Subsequently, the hexahistidine tag was cleaved using thrombin, followed by gel filtration chromatography using a HiLoad 16/60 Superdex75 (GE Healthcare, Chicago, IL, USA) column with 50 mM Tris-HCl, pH 8.0 containing 150 mM NaCl.

#### 4.1.3. C1q

C1q was purified from 40 mL of pooled human serum (Cosmo Bio CO., LTD, Tokyo, Japan) via two-step precipitation at low ionic strength [33]. To 40 mL human serum, 10 mL of 0.1 M Na_2_EDTA solution (pH 7.5) was added to dissociate the C1 complex into its constitutive subunits. To precipitate C1q, 200 mL of 0.01 M Na_2_EDTA (pH 7.5) was added to the solution, which was then incubated on ice for 1 h. The precipitate was collected by centrifugation (12,000× *g* for 30 min, if not specified), washed with 0.04 M Na_2_EDTA (pH 7.5), and collected via centrifugation. The washing step was done twice. The precipitate was dissolved in 10 mL of 0.01 M Na_2_EDTA (pH 5.0) containing 0.75 M NaCl for further purification. The supernatant was collected via centrifugation at 30,000× *g* for 30 min and then dialyzed into 1 L of 0.1 M Na_2_EDTA (pH 5.0) twice. The precipitate was collected via centrifugation, washed in 20 mL of 0.1 M Na_2_EDTA (pH 5.0), and again collected via centrifugation. The washing step was done twice. The collected precipitate was dissolved in 3 mL of 0.01 M Na_2_EDTA (pH 7.5) containing 0.3 M NaCl, and the supernatant was again collected via centrifugation at 30,000× *g* for 30 min. The supernatant contained 0.2 mg/mL C1q.

#### 4.1.4. Cholera Toxin B Subunit

Cholera toxin B subunit (Merck KGaA, Darmstadt, Germany) was purchased in powder form and dissolved at 2.5 mg/mL in phosphate-buffered saline consisting of 137 mM NaCl, 2.7 mM KCl, 8.1 mM Na_2_HPO_4_, and KH_2_PO_4_ (pH 7.4).

### 4.3. NMR Spectroscopy

2D ^1^H–^13^C STD-heteronuclear single-quantum correlation (HSQC) and ^1^H–^13^C HSQC spectral data were acquired for the 2-trimethylsilylethyl derivatives of the GM1 pentasaccharide and the GM2 tetrasaccharide (10 equiv., each) with 50 μM GB2 in phosphate-buffered saline with 99% D_2_O. For the STD experiments [34], spectra were recorded with irradiation at 7.0 ppm for saturation and at 40.0 ppm for reference. NMR spectra were measured using an AVANCE 800 spectrometer (Bruker Co., Billerica, MA, USA) at 25 °C. Data were analyzed using TopSpin 3.6.1(Bruker Co., Billerica, MA, USA) and Sparky.

### 4.4. X-Ray Crystallography

The apo Fc crystals derived from GB2 were obtained in a buffer containing 16% PEG3350, 0.2 M lithium chloride, and 0.1 M Bis-Tris (pH 6.5) after incubation at 20 °C for one week. The Fc fragment cleaved from 7D7 and the B domain of protein A were mixed at a molar ratio of 1:2.5 and then applied to a gel filtration column (Superose 75; GE Healthcare, Chicago, IL, USA) equilibrated with 50 mM Tris-HCl (pH 8.0) containing 150 mM NaCl. Fractions containing the Fc–B domain complex were concentrated to a total protein concentration of 10 mg/mL and used for crystallization. The crystals of Fc in complex with the B domain of protein A were obtained in 60% (*v*/*v*) Tacsimate^TM^ (pH 7.0) incubated at 20 °C for 2 weeks. The crystals were cryoprotected with the crystallization buffer supplemented with 15% glycerol. The apo crystal belonged to space groups *P*2_1_2_1_2_1_ with three Fc dimers per asymmetric unit and diffracted up to a resolution of 2.30 Å. On the other hand, the B-domain-bound Fc crystal belonged to space group *P*4_2_2_1_2, with an asymmetric unit in which one Fc dimer complexed with one B domain, and one Fc monomer formed a homodimer with the crystallographically neighboring Fc molecule and diffracted up to a resolution of 3.30 Å. Diffraction data were integrated and scaled using XDS and AIMLESS [35,36].

The apo and complex crystal structures were solved by the molecular replacement method using the program MOLREP [37] with an apo form of mouse IgG2b-Fc (Protein Data Bank code: 2RGS [38]) as a search model. Manual model fitting to the electron density maps was carried out using COOT [39]. REFMAC5 [40] and phenix.refine [41] were used to refine the crystal structure, and the stereochemical quality of the final model was validated using RAMPAGE [42] (The crystal parameters and refinement statistics are summarized in Supplementary Table S1). The molecular graphics were prepared using PyMOL (http://www.pymol.org/).

### 4.5. PDB Accession Codes

The coordinates and structural factors of the crystal structures of the apo and B-domain-bound forms of mouse IgG2b-Fc have been deposited in the Protein Data Bank under accession numbers 6KRU and 6KRV, respectively.

### 4.6. HS-AFM

Ganglioside (GM1 or GM2) and DOPC were mixed at varying ratios in methanol/chloroform. After solvent evaporation, the residual lipids were suspended at a concentration of 12 mM in 10 mM potassium phosphate buffer (pH 7.2) and then mixed by vortexing. The mixture was sonicated at 70 W for 1 min. The liposome solution of approximately 2 μL was deposited onto a freshly cleaved mica surface and stored at 50 °C in a sealed container to maintain high humidity and prevent surface drying. After a 30-min incubation, the sample surface was thoroughly rinsed with pure water; then, a droplet of antibody solution in phosphate-buffered saline was deposited on the lipid-covered mica surface at a final protein concentration of 10 μg/mL and incubated for 3 min unless otherwise stated. Residual antibodies in the solution were removed by washing. Two molar equivalent of B domain of protein A was added to GB2 before applying to the mica surface. The HS-AFM experiments were conducted three times for each condition. C1q was added at a final concentration of 5–10 μg/mL for observing its interaction with GB2. The mica surface was chemically modified with 0.05% 3-aminopropyltriethoxysilane for observing C1q thereon.

All HS-AFM measurements were carried out using a laboratory-built AFM operated in tapping mode [43,44]. Small cantilevers (BL-AC7DS: Olympus, Tokyo, Japan) with a spring constant of ~0.2 Nm^−1^, a quality factor of approximately 2, and a resonant frequency of ~0.8 MHz (all properties were estimated in water) were used. To achieve a small tip-sample loading force, the free oscillation amplitude of cantilevers was set at 1–2 nm, and the set-point of amplitude for feedback control was approximately 90% of the free amplitude. The HS-AFM experiments were performed at room temperature.

The bound-state dwell time was measured using successive HS-AFM images to estimate the residence time of C1q on the hexameric IgG ring by monitoring the appearance or disappearance of bright spots in the HS-AFM images. All analyses were carried out using laboratory-developed software based on IgorPro 8 (WaveMetrics, Inc., Lake Oswego, OR, USA).

## Supplementary Materials

Supplementary materials can be found at https://www.mdpi.com/1422-0067/21/1/147/s1.

## Abbreviations

IgG	immunoglobulin G
HS-AFM	high-speed atomic force microscopy
HSQC	heteronuclear single-quantum correlation
STD-NMR	saturation transfer difference nuclear magnetic resonance
DOPC	1,2-dioleoyl-sn-glycero-3-phosphocholine
