# Supplementary material for: On-Membrane Dynamic Interplay between Anti-GM1 IgG Antibodies and Complement Component C1q
Source: Int J Mol Sci. 2019 Dec 24;21(1):147. doi: 10.3390/ijms21010147 (PMC6981440; doi:10.3390/ijms21010147)
Supplement: Supplementary file 1 [file ijms-21-00147-s001.zip › supplementary/IJMS-Yanaka-supplementary-191220.docx]

**Supplementary information**

**On-membrane dynamic interplay between anti-GM1 IgG antibodies and complement component C1q**

Saeko Yanaka ^1,2,*^, Rina Yogo ^1,2,*^, Hiroki Watanabe ^1,*^, Yuki Taniguchi ^2^, Tadashi Satoh ^2^, Naoko Komura ^4^, Hiromune Ando ^4^, Hirokazu Yagi ^2^, Nobuhiro Yuki ^5^, Takayuki Uchihashi ^1,3, **^, and Koichi Kato ^1, 2, **^

^1^ Exploratory Research Center on Life and Living Systems (ExCELLS) and Institute for Molecular Science (IMS), National Institutes of Natural Sciences, 5-1 Higashiyama, Myodaiji, Okazaki, Aichi, 444-8787, Japan; yogo@ims.ac.jp, hwatanabe@ims.ac.jp, saeko-yanaka@ims.ac.jp, kkatonmr@ims.ac.jp

^2^ Faculty and Graduate School of Pharmaceutical Sciences, Nagoya City University, 3-1 Tanabe-dori, Mizuho-ku, Nagoya, Aichi, 467-8603, Japan; hyagi@phar.nagoya-cu.ac.jp, tadashisatoh@phar.nagoya-cu.ac.jp, yuuki82540@gmail.com

^3^ Department of Physics, Nagoya University, Furo-cho, Chikusa-ku, Nagoya, Aichi, 464-8602, Japan; uchihast@d.phys.nagoya-u.ac.jp

^4^ Center for Highly Advanced Integration of Nano and Life Sciences (G-CHAIN), Gifu University, Yanagido 1-1, Gifu 501-1193, Japan; komura@gifu-u.ac.jp, hando@gifu-u.ac.jp

^5^ Yuki Clinic, 1-3-7 Johnan, Mito, Ibaraki 310-0803, Japan; gbs.yuki.cidp@gmail.com

* These three authors contributed equally to this work.

** Corresponding author.


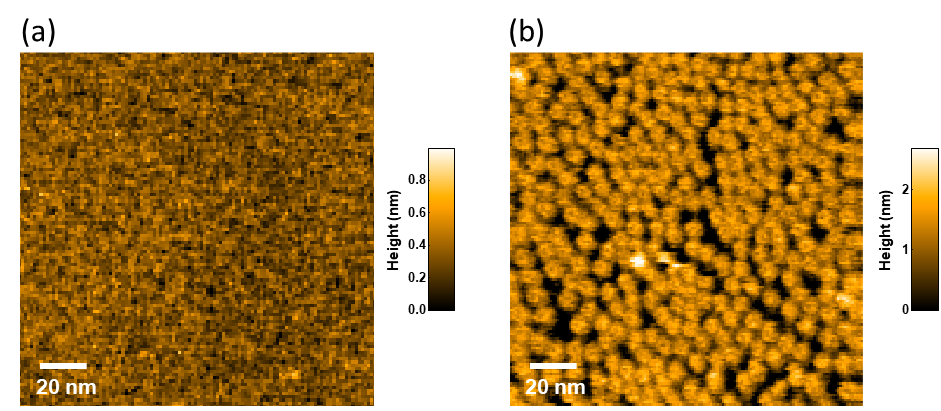


**Supplementary Figure S1**: HS-AFM images of (a) the 50% GM1-incorporated DOPC membranes and (b) cholera toxin B subunit thereon.

**Supplementary Figure S2**: Crystallographic trimer structure of GB2-Fc

Ribbon models of the Fc trimer (yellow, green, and cyan) are shown on the left and right, which are related by a rotation of 180° around a vertical axis.

**Supplementary Figure S3**: Crystal structures of the Fc fragments in the presence and absence of the B domain of protein A. Ribbon model of the Fc complexed with B domain of protein A are shown. Glycans attaching to the Fc domains are indicated as sticks. Molecules A and B are colored in green and cyan, whereas B domain is colored in yellow. The apo GB2-Fc was superimposed on the B domain-bound Fc and shown in gray.

**Supplementary Table S1. Data collection and refinement statistics for the Fc fragments of IgG in the presence and absence of the B domain of protein A**

|  | **Apo Fc** | **Fc/B domain** |
| --- | --- | --- |
| **Crystallographic data** |  |  |
| Space group | *P*2_1_2_1_2_1_ | *P*4_2_2_1_2 |
| Unit cell *a*/*b*/*c* (Å) | 112.6/130.2/134.2 | 145.3/145.3/155.5 |
| α/β/γ (°) | 90.0/90.0/90.0 | 90.0/90.0/90.0 |
| **Data processing statistics** |  |  |
| Beam line | SPring-8 BL44XU | PF-AR NW12A |
| Wavelength (Å) | 0.9000 | 1.0000 |
| Resolution (Å) | 50–2.30 (2.34–2.30) | 50–3.30 (3.53–3.30) |
| Total/unique reflections | 653,358/88,009 | 368,809/25,631 |
| Completeness (%) | 99.9 (97.9) | 99.9 (99.9) |
| *R*_merge_ (%) | 6.3 (101.5) | 8.2 (91.4) |
| *I* / σ (*I*) | 18.3 (1.8) | 26.1 (3.8) |
| **Refinement statistics** |  |  |
| Resolution (Å) | 20.0–2.30 | 20.0–3.30 |
| *R*_work_ / *R*_free_ (%) | 19.8/23.7 | 20.7/25.1 |
| R.m.s.d. from ideal values |  |  |
| Bond lengths (Å) | 0.010 | 0.010 |
| Bond angles (°) | 1.19 | 1.29 |
| Ramachandran plot (%) |  |  |
| Favored | 99.0 | 95.9 |
| Allowed | 0.9 | 4.1 |
| Outliers | 0.1 | 0.0 |

**Supplementary movie legends**

Supplementary Movie S1: HS-AFM movie of GB2 on DOPC membrane.

Captured at a scanning speed of 1 s/frame. Scan area: 200 nm × 200 nm. Pixel size: 150 × 150 pixels^2^. Playing at 5 times recoding speed.

Supplementary Movie S2: HS-AFM movie of assembly of GB2 on DOPC membrane containing 50% GM1.

Captured at a scanning speed of 1 s/frame. Scan area: 200 nm × 200 nm. Pixel size: 150 × 150 pixels^2^. Playing at 5 times recoding speed.

Supplementary Movie S3: HS-AFM movie of an early stage of the GB2 assembly on DOPC membrane containing 50% GM1. The antibody solution deposited on the lipid-covered mica surface was observed without preincubation.

Captured at a scanning speed of 1 s/frame. Scan area: 200 nm × 200 nm. Pixel size: 150 × 150 pixels^2^. Playing at 5 times recoding speed.

Supplementary Movie S4: HS-AFM movie of GB2 on DOPC membrane containing 50% GM2.

Captured at a scanning speed of 1 s/frame. Scan area: 200 nm × 200 nm. Pixel size: 150 × 150 pixels^2^. Playing at 5 times recoding speed.

Supplementary Movie S5: HS-AFM movie of C1q on chemically modified mica surface with 0.05% 3-aminopropyltriethoxysilane.

Captured at a scanning speed of 0.5 s/frame. Scan area: 80 nm × 70 nm. Pixel size: 75 × 60 pixels^2^. Playing at 2 times recoding speed.

Supplementary Movie S6: HS-AFM movie of C1q on DOPC membrane containing 50% GM1.

Captured at a scanning speed of 0.2 s/frame. Scan area: 120 nm × 95 nm. Pixel size: 120 × 95 pixels^2^. Playing at 2 times recoding speed.

Supplementary Movie S7: HS-AFM movie of C1q on DOPC membrane containing 50% GM1 with the GB2 antibody.

Captured at a scanning speed of 0.2 s/frame. Scan area: 140 nm × 100 nm. Pixel size: 85 × 65 pixels^2^. Playing at 2 times recoding speed.
